# The Dysarthria Impact Profile: A Preliminary French Experience with Parkinson's Disease

**DOI:** 10.1155/2013/403680

**Published:** 2013-05-23

**Authors:** Alban Letanneux, Margaret Walshe, François Viallet, Serge Pinto

**Affiliations:** ^1^Aix-Marseille Université, CNRS, Laboratoire Parole et Langage (LPL), UMR 7309, BP 80975, 13604 Aix-en-Provence Cedex 1, France; ^2^Department of Clinical Speech and Language Studies, Trinity College Dublin, 7-9 South Leinster Street, Dublin 2, Ireland; ^3^Service de Neurologie, Centre Hospitalier du Pays d'Aix, Avenue des Tamaris, 13616 Aix-en-Provence Cedex 1, France

## Abstract

This preliminary study aimed to adapt the Dysarthria Impact Profile (DIP) in French and to confirm its relevance for the assessment of the psychosocial impact of dysarthria in Parkinson's disease (PD). The DIP scale was administered to 10 people with PD and 10 age-matched control subjects. The DIP psychometric properties were calculated (discriminant validity, internal consistency, and concurrent validity), notably by using the Voice Handicap Index (VHI) for interscale comparisons. The French version of the DIP discriminated people with PD from control subjects (*χ* ^2^ test, *P* < 0.05). Good internal consistency was observed in both populations (Cronbach's *α* = 0.93 for PD people and *α* = 0.76 for control subjects). The DIP was highly correlated with the VHI (Spearman's *ρ* = −0.70, *P* < 0.01), confirming the external validity of the scale. There was no direct relationship between PD speech and quality of life as assessed by the Parkinson's Disease Questionnaire-39 (PDQ-39). Our preliminary data suggest that the French version of the DIP has the potential to make a useful contribution for the assessment and outcome management in acquired dysarthria for both clinicians and researchers.

## 1. Introduction

Improving quality of healthcare and encouraging clinicians to adopt a more holistic approach to the assessment and treatment of patients were significant contributions of the International Classification of Functioning Disability and Health (ICF), promoted by the World Health Organization (WHO) during the 2001 international conference in Geneva [1] to the field of speech sciences. Since the adoption of this framework, considering patients' personal feelings regarding physical, psychological, and social domains has received increasing interest over the last decade. Classical assessment procedures now aim at including evaluations of quality of life and well-being in populations with communication impairments [2]. However, the few tools available for the investigation of the psychological and social impact of oral communication deficits mainly focus on voice and speech production disorders [3–6].

Hypokinetic dysarthria in Parkinson's disease (PD) is a motor speech disorder that arises as a consequence of a neurodegenerative process. Around 70% of people with PD are affected to some degree by voice and speech impairment [7], leading one to consider that communication impairment is highly prevalent and debilitating in this population. Indeed, people with PD are less likely to participate in conversations, or to have confidence in social interactions [8]. Several studies suggest that as PD progression is associated with a growing discomfort in verbal communication, there is an important negative alteration to social life [9–11]. Capturing the impact of dysarthria on the person with PD is not straightforward. While there are many clinical and instrumental ways to evaluate dysarthria, the person's own experience of his/her communicative limitations has been long neglected. Even if dedicated self-reporting questionnaires for the assessment of voice and speech difficulties arising from dysarthria are available [12–14], scales examining the impact of such difficulties on daily-living activities are scarce. To address this limitation, the Dysarthria Impact Profile (DIP) has been proposed as an alternative for the assessment of the psychosocial impact of dysarthria on speakers [15]. Whereas the gold standard dysarthria questionnaires mainly assess speech and/or voice parameters (e.g., acoustics, articulation), the original approach of the DIP is to focus on the impact of speech deficits', specifically the psychosocial impact of the speech disorder on the communicative participation from the speaker's perspective.

The term “psychosocial impact” is multidimensional [16, 17], defined as “*the psychological and social consequences of a motor speech disorders with quality of life, subjective well-being, and societal participation, viewed predominantly as consequence or factors that contribute to psychosocial impact*” [2]. The DIP scale was designed using data from in-depth interviews with people presenting with non-congenital dysarthria [18, 19], drawing also from earlier accounts from other researchers [18, 20–23] and personal accounts of individuals with dysarthric speech [24–26]. From these data, forty-eight items were drawn up and divided into five specific topic areas: (1) the effect of dysarthria on me as a speaker, (2) accepting my dysarthria, (3) how I feel others react to my speech, (4) how dysarthria affects my communication with others, and (5) dysarthria relative to other worries and concerns [15]. This scale has been used in studies examining the psychosocial impact of dysarthria from the speaker's perspective [23, 27, 28].

The DIP was devised in English and is used with English speaking populations. The main goal of this study was to translate the DIP into French and assess its relevance in French for the description of the psychosocial impact of dysarthria in PD. We explored the discriminant validity, (i.e., the comparison between the PD population scores with the scores from control subjects); the concurrent validity of the DIP, by calculating its correlation with other self-reporting questionnaires that aimed at evaluating associated constructs of voice handicap and quality of life; and the relationship between the negative impact of PD speech and negative quality of life.

## 2. Material and Methods

### 2.1. Participants

Ten people with PD (6 males and 4 females) and under medication participated in the study. They were recruited by a movement disorders' neurologist (F.V.) at the Neurology Department of the Aix-en-Provence Hospital (Centre Hospitalier du Pays d'Aix), where they attended outpatient clinics. The mean (±SD) age of the participant sample was 68.6 ± 12.3 years (age range: from 47 to 84 years). The mean (±SD) disease duration was 4.7 ± 3.6 years (range: from 4 months to 9 years). The selection criteria included patients diagnosed with idiopathic PD, no cognitive impairment, no history of hearing impairment, and no previous speech therapy rehabilitation. The patients recruited underwent an examination using part III of the Unified Parkinson's Disease Rating Scale, UPDRS [29], which assessed the global motor state of the patients. The mean (±SD) UPDRS III score *on* medication was 14.1 ± 7.2. The cognitive state of the patients was evaluated using either the Mattis Dementia Rating Scale [30] or the Mini-Mental State Examination [31]. None of the participants had evidence of any cognitive impairment.

Ten age-matched healthy control subjects (4 males and 6 females) were recruited from the experimenters' personal contacts to participate in the study. The mean (±SD) age of the control group was 72.4 ± 3.9 years (age range: from 70 to 83 years). None of the control subjects had history of any hearing or cognitive impairment or other neurologic or psychiatric diseases. Demographics and characteristics of people with PD and control participants are summarized in Table 1.

### 2.2. Assessment Measures

Two valid and reliable assessment measures (the Voice Handicap Index, VHI, and the Parkinson's Disease Questionnaire, PDQ-39) were used along with the DIP. All the assessments were administered in one day. Regarding the people with PD, three participants completed the scales with the help of an experimenter (A.L.), since they presented with writing difficulties; the remainder completed the scales independently. All control participants completed the scales independently at home.

#### 2.2.1. Dysarthria Impact Profile (DIP)

Items from the original questionnaire were translated into French by A.L. & S.P. and compared with the original items by a bilingual linguistics researcher. The final translated version integrated all the translation adjustments. In the first 4 sections of the scale, the person with PD was asked to rate statements in each section using a five-point scale (“strongly agree,” “agree,” “not sure,” “disagree,” and “strongly disagree”). In order to test the responder reliability (i.e., the participant answer congruence or responder consistency), the DIP scale incorporated 2 similar statements in each section, differently formulated. The DIP uses positively and negatively worded statements. In the positively worded statements, “strongly agrees” answers receive a score of 5 and “strongly disagree” answers receive a score of 1. Reversely, in negatively worded statement, “strongly agrees” answers receive a score of 1 and “strongly disagree” answers receive a score of 5. In the fifth section, people were asked to list and rank (from 1 = most worry to 5 = least concern) their five main worries, including speech impairment. The DIP could be completed by the person him/herself or with assistance. All answers were added to obtain a global impact score; the lower the score, the higher the level of impact. 

#### 2.2.2. Voice Handicap Index (VHI)

The VHI [3] is often considered as the *gold standard* for the evaluation of voice self-perception [32]. It includes 30 items split into three domains: physical, functional, and emotional. It has been previously translated into French and validated with a French population [33]. Each item is scored from 0 to 4 (“never,” “almost never,” “sometimes,” “almost always,” and “always”); the higher the score, the higher the degree of perceived handicap.

#### 2.2.3. Parkinson's Disease Questionnaire (PDQ-39)

The PDQ-39 [34] is a global and PD-specific quality of life questionnaire, also available in French [35]. The scale consists in 39 items allowing for the determination of an overall quality-of-life score examining 8 specific domains: mobility, activities of daily living, emotional well-being, stigma, social support, cognition, communication, and bodily discomfort. Each item is scored from 0 (normal) to 4 (maximum disturbance); the higher the score, the higher the impairment of quality of life.

### 2.3. Statistical Analyses

Statistical analyses (R Development Core Team, http://www.r-project.org/) were carried out in order to estimate the psychometric properties of the French version of the DIP in our preliminary set of data. Since the DIP scale is nominal, *χ* ^2^ test was used to evaluate the ability of distinguishing people with PD from controls (discriminant validity). Regarding the VHI, which is an ordinal scale, Wilcoxon ranked test was performed. Internal consistency of the DIP was assessed by calculating Cronbach's *α* coefficient for each population. Responder reliability was tested by measuring correlations between connected items within each of the first 4 sections (Spearman's *ρ* coefficient). Concurrent validity was assessed by correlating the DIP and VHI scores (Spearman's *ρ* coefficient).

## 3. Results and Discussion

### 3.1. DIP Main Effects and Discriminant Validity

As it can be seen in Table 2, the people with PD obtained lower scores on the DIP than control subjects, suggesting a high level of psychosocial impact of dysarthria in this population. The French version of the DIP was able to discriminate between control subjects and people with PD (*χ* ^2^ = 176.6, df = 4, *P* < 0.05), as did the VHI total score (Wilcoxon *W* = 78.5, *P* < 0.05). In Section E of the DIP (“*Dysarthria relative to other worries and concerns*”), speech was the main concern for only 1 out of the 10 PD participants and an important worry for 5 of them (50%). The remaining four people with PD were slightly or least concerned about their speech.

### 3.2. Internal Consistency

Internal consistency, (i.e., how well all items in a scale correlate with each other and follow the same trend) was assessed using Cronbach's *α* coefficient. An adequate consistency is considered with a coefficient of at least 0.70. Thus, internal consistency was confirmed for the DIP total scores for both the PD (*α* = 0.93) and control (*α* = 0.76) populations. Internal consistencies were also confirmed for the DIP subsections A (*α* = 0.85), B (*α* = 0.72), C (*α* = 0.87), and D (*α* = 0.83) for the PD patients.

### 3.3. Responder Reliability

Responder reliability was tested by measuring correlations between 2 connected items within each subsection (Spearman's *ρ* correlation), sections A (“*The effect of dysarthria on me as a speaker*”), B (“*Accepting my dysarthria*”), and D (“*How dysarthria affects my communication with others* of the scale”) displayed statistically significant correlations (*ρ* = 0.76, *ρ* = 0.59, and *ρ* = 0.72, resp., *P* < 0.01), whereas the connected items of section C (“*How I feel others react to my speech*”) did not show any correlation (*ρ* = 0.39, *P* = 0.08).

### 3.4. Concurrent Validity of the DIP

**Figure 1:**
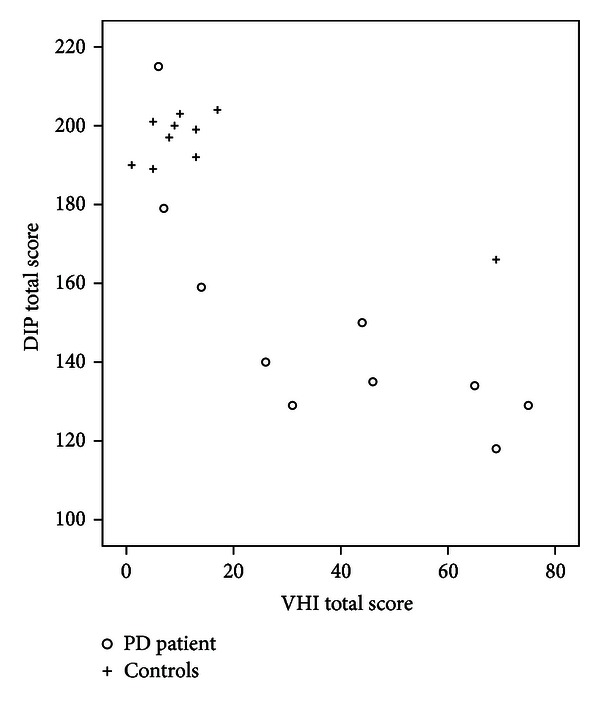
Voice Handicap Index (VHI) versus Dysarthria Impact Profile (DIP) total scores for the 20 participants (10 people with PD and 10 control subjects).

Although the DIP, VHI, and PDQ-39 levels of measurements are different (nominal for the DIP, ordinal for the VHI and PDQ-39), we thought important to have an idea of the construct validity by testing, nevertheless, correlation analyses between the scales' total scores. As displayed in Figure 1, correlations between the DIP and the VHI were high for both the people with PD and the control subjects (Spearman's *ρ* = −0.70; *P* < 0.01). Furthermore, in the PD group, the total DIP score correlated significantly with the “Functional” sub-section (the impact of voice disorders on daily living activities) of the VHI scale (*ρ* = −0.82, *P* < 0.01). This was not the case with the PDQ-39 total score (*ρ* = −0.41, *P* = 0.23).

### 3.5. Discussion Points

Due to the small number of participants in the present study, our findings still have to be considered as preliminary. Despite that fact, our data were able to demonstrate that (1) the French DIP was able to discriminate people with PD from control subjects, (2) the DIP was highly correlated with the VHI, and (3) no direct relationship between the DIP and quality of life as assessed by the PDQ-39 was displayed. This last point should be expected as the DIP does not assess quality of life and the authors of the DIP view quality of life and psychosocial impact as separate constructs [19].

Speech and voice impairments are frequent in patients with PD. PD speech manifesting itself most typically as a hypokinetic dysarthria displays a combination of respiratory, laryngeal, resonance, and supralaryngeal articulatory deficits [36, 37]. Dysphonia in PD involves monotony of pitch and loudness, a breathy and harsh voice, which represents crucial parameters altering speech intelligibility and, as a consequence, communication ability. This might account for the strong correlation found between the DIP and VHI scales. Methodological constraints did not allow us to acquire neither any acoustical recordings nor any formal assessment of the speech disorder severity. Further experiments will need to take into account this aspect in order to explore the relationship between the DIP and the severity of the speech impairment. Moreover, this will be particularly of interest when assessing different types of dysarthria, as carried out, for example, regarding the validation of the French version of the Speech Handicap Index [14].

The original DIP questionnaire was administered to 31 people with dysarthria, 10 of whom had PD [15]. We performed the present exploratory study with the same number of people with PD. In our group, the mean DIP score was calculated to be 148.8 (cf. Table 2), which is similar to that provided in the original study (144.9). Both English and French versions of the DIP show good internal consistency, arguing a reasonable similarity of our French version with the original one. However, the lack of intraindividual congruence of the 2-connected answers in section C we found in the French version might result from the design of the DIP, particularly the alternation between positive and negative worded statements. In fact, this shift may also imply difficulties for both patients and control subjects when completing the scale, leading to possible incongruent responses. This was not the case with the original version, leading us to consider the need for some amendments in the French version of the scale. Additionally, the completion time of the DIP French version was rather long, more than 30 minutes on average for PD patients. Difficulties when answering might also be due to the visual form of the DIP. Changes are recommended in order to improve both reading and recording responses and in order to shorten the completion time for patients. These represent practical issues that will be taken into account in a further amended format of both English and French versions of the scale. Work on this is currently underway.

Some reliability and validity has been established with the French DIP used in the present experiment. However, further validation needs to be performed in order to confirm the psychometric properties demonstrated in our preliminary results. To do so, the amended version of the French DIP will need to be administered on a larger sample of people with dysarthria, covering a range of different aetiologies. Due to the fact that control subjects may also present with speech alteration associated with age, as suggested by the performance of one control (cf. Table 2 and Figure 1), a larger number of controls covering a range of ages will also be required. Test/reretest reliability and responsiveness to change will have to be considered as well. Work on these aspects is underway in the English version. Regarding specifically the French version, a back-to-back translation procedure should be planned for the updated version of the scale.

## 4. Conclusions

While some behavioural treatments for speech in PD might have predictable beneficial impacts [38], pharmacological and/or neurosurgical treatments have relatively limited and variable effects on PD speech [39]. There is a need to consider treatments beyond the level of impairment thereby justifying the use of a self-administered questionnaire to measure the psychosocial impact of dysarthria from the perspective of the speaker. This is particularly of interest since this kind of self-assessment may help the patient realise his/her difficulties and lead him/her to take part with the clinician in therapeutic work. The DIP is part of a range of new self-assessments, including the Speech Handicap Index [12, 14] and the French *Parole Handicap Index* [13], all aiming at producing “*a comprehensive picture of speech impairment*” [14]. Our preliminary data suggest that the French version of the DIP may have the potential to make a useful contribution to outcome management in acquired dysarthria for both clinicians and researchers.

## Figures and Tables

**Table 1:** Demographics and characteristics of people with PD and control participants.

People with PD^a^	Gender	Age (years)	Disease duration (years)	UPDRS score on medication	Controls^b^	Gender	Age (years)
P1	Male	55	1.5	11	C1	Female	70
P2	Female	76	6.6	8	C2	Female	70
P3	Male	75	2.0	15	C3	Female	72
P4	Male	56	6.8	25	C4	Male	72
P5	Female	78	9.0	20	C5	Female	72
P6	Female	72	8.0	4	C6	Male	74
P7	Female	84	9.3	Missing data	C7	Female	83
P8	Male	47	1.0	13	C8	Male	70
P9	Male	64	0.3	8	C9	Male	70
P10	Male	79	2.0	23	C10	Female	71

Mean ± SD		68.6 ± 12.3	4.7 ± 3.6	14.1 ± 7.2			72.4 ± 3.9

^a^The selection criteria included patients diagnosed with idiopathic PD, no cognitive impairment, no history of hearing impairment, and no previous speech therapy rehabilitation. The cognitive state of the patients was evaluated using either the Mattis Dementia Rating Scale [30] or the Mini-Mental State Examination [31]. None of the participants had any evidence of cognitive impairment. ^b^None of the control subjects had any history of hearing or cognitive impairment or other neurologic or psychiatric disease.

**Table 2:** Total and mean scores from self-assessments.

People with PD	DIP^a^ (score/225)	VHI^b^ (score/120)	PDQ-39^b^ (score/156)	Controls	DIP	VHI
P1	159	14	41	C1	166	69
P2	215	6	18	C2	189	5
P3	118	69	58	C3	200	9
P4	129	31	27	C4	192	13
P5	179	7	37	C5	201	5
P6	140	26	38	C6	204	17
P7	134	65	65	C7	190	1
P8	150	44	86	C8	197	8
P9	135	46	19	C9	203	10
P10	129	75	95	C10	199	13

Mean ± SD	148.8 ± 29.0	38.3 ± 25.6	48.4 ± 26.85		194.1 ± 11.1	15 ± 19.5

^a^The lower the score, the higher the impact; ^b^the higher the score, the higher the impairment.

## References

[1] International Classification of Functioning (ICF) (2001). *Disability and Health*.

[2] Walshe M, Lowit A, Kent RD (2010). The psychosocial impact of acquired motor speech disorders. *Assessment of Motor Speech Disorders*.

[3] Jacobson BH, Johnson A, Grywalski C (1997). The Voice Handicap Index (VHI): development and validation. *American Journal of Speech-Language Pathology*.

[4] Hogikyan ND, Sethuraman G (1999). Validation of an instrument to measure voice-related quality of life (V-RQOL). *Journal of Voice*.

[5] Ma EPM, Yiu EML (2001). Voice activity and participation profile: assessing the impact of voice disorders on daily activities. *Journal of Speech, Language, and Hearing Research*.

[6] Deary IJ, Wilson JA, Carding PN, MacKenzie K (2003). VoiSS: a patient-derived voice symptom scale. *Journal of Psychosomatic Research*.

[7] Angeli S, Marchese R, Abbruzzese G (2003). Tilt-table test during transcranial Doppler monitoring in Parkinson’s disease. *Parkinsonism and Related Disorders*.

[8] Fox CM, Ramig LO (1997). Vocal sound pressure level and self-perception of speech and voice in men and women with idiopathic Parkinson disease. *American Journal of Speech-Language Pathology*.

[9] Jenkinson C, Peto V, Fitzpatrick R, Greenhall R, Hyman N (1995). Self-reported functioning and well-being in patients with Parkinson’s disease: comparison of the short-form wealth survey (SF-36) and the Parkinson’s Disease Questionnaire (PDQ-39). *Age and Ageing*.

[10] Martinez-Martin P (1998). An introduction to the concept of “quality of life in Parkinson’s disease”. *Journal of Neurology*.

[11] Kuopio AM, Marttila RJ, Helenius H, Toivonen M, Rinne UK (2000). The quality of life in Parkinson's disease. *Movement Disorders*.

[12] Rinkel RN, Verdonck-de Leeuw IM, van Reij EJ, Aaronson NK, Leemans R (2008). Speech Handicap Index in patients with oral and pharyngeal cancer: better understanding of patients’ complaints. *Head and Neck*.

[13] Fichaux-Bourin P, Woisard V, Grand S, Puech M, Bodind S (2009). Validation d'un questionnaire d’auto-évaluation de la parole (Parole Handicap Index). *Revue de Laryngologie Otologie Rhinologie*.

[14] Degroote G, Simon J, Borel S, Crevier-Buchman L (2011). The French version of Speech Handicap Index: validation and comparison with the Voice Handicap Index. *Folia Phoniatrica et Logopaedica*.

[15] Walshe M, Peach RK, Miller N (2009). Dysarthria Impact Profile development of a scale to measure psychosocial effects. *International Journal of Language and Communication Disorders*.

[16] Parr S (2001). Psychosocial aspects of aphasia: whose perspectives?. *Folia Phoniatrica et Logopaedica*.

[17] Brumfitt S (2006). Psychosocial aspects of aphasia: speech and language therapists’ views on professional practice. *Disability and Rehabilitation*.

[18] Walshe M (2002). *You have no idea what it is like not to be able to talk’. Exploring the impact and experience of acquired neurological dysarthria from the speaker’s perspective [Unpublished Ph.D. thesis]*.

[19] Walshe M, Miller N (2011). Living with acquired dysarthria: the speaker’s perspective. *Disability and Rehabilitation*.

[20] Antonius K, Beukelman D, Reid R, Robin D, Yorkston K, Beukelman D (1996). Communication disability of Parkinson’s disease: perceptions of dysarthric speakers and their primary communication partners. *Disorders of Motor Speech: Assessment, Treatment, and Clinical Characterization*.

[21] Yorkston KM, Strand EA, Kennedy MRT (1996). Comprehensibility of dysarthric speech: implications for assessment and treatment planning. *American Journal of Speech-Language Pathology*.

[22] Miller N, Noble E, Jones D, Burn D (2006). Life with communication changes in Parkinson’s disease. *Age and Ageing*.

[23] Dickson S, Babour RS, Brady M, Alexander M, Paton G (2008). Patients’ experiences of disruptions associated with post-stroke dysarthria. *International Journal of Language & Communication Disorders*.

[24] Bauby D (1997). *The Diving Bell and the Butterfly*.

[25] Cant R (1997). Rehabilitation following a stroke: a participant perspective. *Disability and Rehabilitation*.

[26] McCrum R (1998). *My Year off: Rediscovering Life After a Stroke*.

[27] Mackenzie C, Lowit A (2007). Behavioural intervention effects in dysarthria following stroke: communication effectiveness, intelligibility and dysarthria impact. *International Journal of Language and Communication Disorders*.

[28] Griffiths S, Barnes R, Britten N, Wilkinson R (2011). Investigating interactional competencies in Parkinson’s disease: the potential benefits of a conversation analytic approach. *International Journal of Language & Communication Disorders*.

[29] Fahn S, Elston R, Fahn S, Marsden CD, Goldstein M, Calne DB (1987). Unified Parkinson’s Disease Rating Scale. *Recent Developments in Parkinson’s Disease. Vol. II*.

[30] Mattis S, Bellack L, Karasu TB (1976). Mental status examination for organic mental syndrome in the elderly patient. *Geriatrics Psychiatry: A Handbook for Psychiatrists and Primary Care Physicians*.

[31] Folstein MF, Folstein SE, McHugh PR (1975). Mini mental state. A practical method for grading the cognitive state of patients for the clinician. *Journal of Psychiatric Research*.

[32] Kasper C, Schuster M, Psychogios G (2011). Voice Handicap Index and voice-related quality of life in small laryngeal carcinoma. *European Archives of Oto-Rhino-Laryngology*.

[33] Woisard V, Bodin S, Puech M (2004). The Voice Handicap Index: impact of the translation in French on the validation. *Revue de Laryngologie Otologie Rhinologie*.

[34] Peto V, Jenkinson C, Fitzpatrick R, Greenhall R (1995). The development and validation of a short measure of functioning and well being for individuals with Parkinson’s disease. *Quality of Life Research*.

[35] Auquier P, Sapin C, Ziegler M (2002). Validation en langue française d’un questionnaire de qualité de vie dans la maladie de Parkinson: le Parkinson’s Disease Questionnaire-PDQ 39. *Revue Neurologique*.

[36] Darley FL, Aronson AE, Brown JR (1969). Differential diagnostic patterns of dysarthria. *Journal of Speech and Hearing Research*.

[37] Duffy JR, Duffy JR (2005). Defining, understanding, and categorizing motor speech disorders. *Motor Speech Disorders—Substrates, Differential Diagnosis, and Management*.

[38] Yorkston KM, Hakel M, Beukelman DR, Fager S (2007). Evidence for effectiveness of treatment of loudness, rate, or prosody in dysarthria: a systematic review. *Journal of Medical Speech-Language Pathology*.

[39] Pinto S, Ozsancak C, Tripoliti E, Thobois S, Limousin-Dowsey P, Auzou P (2004). Treatments for dysarthria in Parkinson’s disease. *Lancet Neurology*.

